# Advancing precision antiviral therapy: The value, challenges, and prospects of a novel antiviral crRNA screening platform

**DOI:** 10.1016/j.omtn.2025.102736

**Published:** 2025-10-23

**Authors:** Shuaiyin Chen, Jinzhao Long, Guangcai Duan

**Affiliations:** 1 Department of Epidemiology, College of Public Health, Zhengzhou University, Zhengzhou 450001, China

## Main text

A promising study by Niu et al., published in *Molecular Therapy Nucleic Acids*, presented a rapid and efficient screening platform utilizing the bioinformatics tool CaSilico in conjunction with CRISPR-Cas13 *in vitro* detection to identify highly effective antiviral crRNAs.^1^ The authors successfully demonstrated its utility by targeting both SARS-CoV-2 and dengue virus (DENV), with one candidate crRNA (E−2330) achieving over 90% suppression across all four DENV serotypes. These findings not only provide an efficient screening paradigm for antiviral CRISPR therapies but also highlight the platform’s potential in addressing rapidly mutating RNA viruses. However, the predictive accuracy of the platform still requires further improvement, and its *in vivo* efficacy and long-term safety necessitate additional experimental validation.

RNA viruses represent a major class of pathogens that pose a threat to human health, including SARS-CoV-2, DENV, the tumor-inducing hepatitis C virus (HCV), and lethal encephalitis viruses.^2^^,^^3^ Public health incidents caused by these viruses have had a profound global impacts. The high mutation rate and rapid evolution of RNA viruses present significant challenges for treatment and prevention.^4^ Traditional antiviral strategies, such as vaccines and specific antiviral drugs, have shown success in controlling certain viruses. However, they still encounter limitations, such as slow response times, lengthy development cycles, and reduced efficacy against emerging variants. The prolonged development timeline implies that during this period, millions of people may be infected, and the virus may accumulate critical mutations, potentially giving rise to new variants. This not only exacerbates the risk of outbreak spread but may also render newly developed interventions obsolete. Therefore, there is an urgent need to develop broad-spectrum, rapid, and adaptive antiviral technologies—an undisputed priority in the field of infection prevention and control.

In recent years, nucleic acid-targeting technologies such as RNA interference (RNAi), antisense oligonucleotides (ASOs), and CRISPR systems have opened new avenues for antiviral therapy. Among these, the CRISPR-Cas system is distinguished by its powerful programmability, high specificity, and efficient cleavage of target nucleic acids.^5^ Specifically, the CRISPR-Cas13 system, which directly targets and cleaves RNA viral genomes, has been shown to effectively suppress viral replication in multiple model systems.^6^^,^^7^ Nevertheless, a major obstacle to broader application is the need to rapidly and accurately identify highly efficient and specific crRNAs from a vast number of candidate sites in viral genomes. Conventional screening methods depend on empirical design, low-throughput experimental validation, and limited computational prediction tools. These approaches are not only time-consuming and labor-intensive but also inadequate for keeping pace with the rapid evolution of viruses.

To address this limitation, the study integrated the bioinformatics tool CaSilico with CRISPR-Cas13-based *in vitro* detection. Utilizing the mechanistic similarities between CRISPR-based nucleic acid detection and its antiviral targeting strategies, the authors established a correlation between the fluorescence signal intensity from CRISPR nucleic acid testing and the antiviral efficacy of CRISPR-mediated interference. This correlation enabled the development of an efficient and rapid screening method for identifying highly active antiviral crRNAs. Subsequently, the study carried out verification and exploration in two key aspects. First, a rapid screening platform for antiviral crRNA targets was successfully established based on a SARS-CoV-2 reporter gene system. The findings not only validated the feasibility of integrating the CaSilico bioinformatic approach with CRISPR-Cas13-based *in vitro* detection for screening anti-RNA virus crRNAs, but also demonstrated that this platform significantly reduces the screening duration and enhances the overall efficiency of identifying antiviral crRNAs and their respective targets. Second, using this platform, a crRNA targeting the DENV E protein, (designated as E−2330), was identified. Experimental results demonstrated that this crRNA effectively inhibited DENV replication in both Vero and HepG2 cells, with an efficiency exceeding 90%, and exhibited antiviral activity against all four serotypes of DENV. Given the current lack of effective treatments for most RNA virus infections, this platform holds considerable potential for screening antiviral crRNAs against other RNA viruses, offering broad application prospects. Overall, the study presents a novel approach to crRNA screening that advances antiviral research.

The primary significance of this work lies in its methodological innovation. By offering a faster, more cost-effective, and scalable approach to identifying targets, it reduces the obstacles to developing CRISPR-based treatments for a variety of RNA viruses. Nevertheless, several important limitations should be noted. Validation was performed only in cellular models, leaving open key questions regarding *in vivo* delivery efficiency, potential off-target effects, and the immunogenicity of the CRISPR-Cas13 system in animal models. Moreover, the correlation between fluorescence signal intensity (used for *in vitro* screening) and actual antiviral efficacy within cells requires further validation across multiple virus genera and cell models. These uncertainties in translatability and the gap between *in vitro* and *in vivo* performance represent central bottlenecks hinder the transition of this technology from basic research to clinical applications.

Looking forward, this platform demonstrates potential beyond being merely a screening tool; it could become a key component of future antiviral preparedness strategies. One can envision its integration with machine learning refining the prediction of candidate crRNAs, while advanced delivery technologies, such as lipid nanoparticles (LNPs) and adeno-associated viruses (AAVs), may enable the rapid generation of preclinical candidates against emerging pandemic threats. If current limitations are addressed through further research, this approach could pave the way for a more proactive response paradigm—potentially allowing for the timely development of sequence-specific antivirals early in an outbreak, before widespread global transmission occurs.

## Declaration of interests

I hereby declare that there are no competing financial interests or personal relationships that could have influenced the content or opinions expressed in this commentary. The authors of the original article have not had any prior collaborative or supervisory relationship with me, and this review was conducted impartially based solely on the scientific merit of the work.

## References

[1] Niu M., Dong Z., Yu L., Dong X., An J., Han Y., Yan Y., Yi W., Sun Y., Li H. (2025). Anti-RNA virus crRNA targets efficient screening platform based on bioinformatics and CRISPR detection. Mol. Ther. Nucleic Acids.

[2] Hu B., Guo H., Zhou P., Shi Z.L. (2021). Characteristics of SARS-CoV-2 and COVID-19. Nat. Rev. Microbiol..

[3] Bhatt S., Gething P.W., Brady O.J., Messina J.P., Farlow A.W., Moyes C.L., Drake J.M., Brownstein J.S., Hoen A.G., Sankoh O. (2013). The global distribution and burden of dengue. Nature.

[4] Domingo E., Holland J.J. (1997). RNA virus mutations and fitness for survival. Annu. Rev. Microbiol..

[5] Mohanraju P., Makarova K.S., Zetsche B., Zhang F., Koonin E.V., van der Oost J. (2016). Diverse evolutionary roots and mechanistic variations of the CRISPR-Cas systems. Science.

[6] Abbott T.R., Dhamdhere G., Liu Y., Lin X., Goudy L., Zeng L., Chemparathy A., Chmura S., Heaton N.S., Debs R. (2020). Development of CRISPR as an Antiviral Strategy to Combat SARS-CoV-2 and Influenza. Cell.

[7] Mahas A., Aman R., Mahfouz M. (2019). CRISPR-Cas13d mediates robust RNA virus interference in plants. Genome Biol..

